# Structural optimization of pit bionic drip irrigation emitter to improve hydraulic performance and anti-clogging performance

**DOI:** 10.1371/journal.pone.0334698

**Published:** 2025-10-24

**Authors:** Zhen Zhao, Tianyu Xu, Yanru Su, Sanlin Bao, Qiuyue Yu

**Affiliations:** 1 School of Hydraulic and Electric Power, Heilongjiang University, Harbin, China; 2 Heilongjiang Academy of Black Soil Conservation and Utilization, Harbin, China; Ardakan University, IRAN, ISLAMIC REPUBLIC OF

## Abstract

The blockage inside the flow channel of drip irrigation emitters is a key issue that restricts their usability. The low-speed vortex zone that exists in traditional channel design is the core reason. This study designed four different structures of pit bionic drip irrigation emitters based on the principles of plant bionics. The computational fluid dynamics (CFD) numerical simulation method was adopted and the flow velocity and turbulence energy of four structures were analyzed. The discrete element method (DEM) was combined to study the motion trajectory of 0.1 mm sand particles. The results indicate that schemes 1, 2, and 4 all have significant low-speed vortices in the return water zone (D zone). The situation in scheme 3 is relatively mild and the probability of sand particles depositing in the channel decreases. The flow channel structure was further optimized based on the original foundation and eight types of sand particles with different sizes were selected for anti clogging experiments. The relative traffic of the optimized model in the third and fourth stages was 13.34% and 14.51% higher. In terms of the sensitive particle size causing blockage, the maximum allowable particle size of the optimized flow channel structure was nearly twice that of scheme 3. When the particle size was 0.120, 0.165, 0.187, 0.212, and 0.245 mm, the sedimentation rate was reduced by an average of 58.02%. This study confirms that optimized drip irrigation emitters have better anti clogging performance under multiple particle size drip irrigation conditions.

## Introduction

As the core technology in the field of water-saving irrigation, the drip irrigation system plays a pivotal role with its advantages of water saving, fertilizer saving, high yield and high irrigation efficiency [1,2]. The drip irrigation emitters were the most important component of drip irrigation systems [3]. Its flow channel structure was complex and the size was small. The sand particles in irrigation water were prone to sedimentation inside the channel, which will lead to serious blockage of the emitter and directly affect the service life and irrigation efficiency of the whole drip irrigation system [4,5]. So far, many scholars have studied the blockage problem of drip irrigation emitters from multiple perspectives, and certain results have been achieved. Oliveira et al. [6] explained that the types and structural parameters of the flow channel had a significant impact on the clogging of drip irrigation emitters. Baghel et al. [7] used computational fluid dynamics to elucidate the mechanism of the influence of channel structure on the water flow energy consumption and hydraulic performance of drip irrigation emitters. Yu et al. [8] used the particle tracking velocimetry testing platform to visualize the motion characteristics of sand particles in the channel, which showed that appropriate structural parameters were beneficial for sand particles to migrate out of the entire channel and reduce the occurrence of blockages. The simplest and most effective way to reduce sand blockage was to optimize the flow channel structure [9,10].

The main factor causing physical blockage of drip irrigation emitters are the deposition of small sand particles. Some scholars have explained the clogging mechanism of drip irrigation emitters and proposed relevant solutions [11,12]. Liu et al. [13] found that the influence of particle size on clogging was not monotonously increasing or decreasing, which was related to the sensitivity of drip irrigation emitters to particle size. Ait-Mouheb et al. [11] studied the influence of hydrodynamic conditions on clay particle deposition and biofilm development in emitters by an optical method. The results showed that the physical and biological clogging were observed to occur mainly in the inlet channel and initial vortex zones, which were characterized by lower mean velocity and turbulent kinetic energy values. Pereira et al. [14] proposed a clogging test protocol for evaluating the sensitivity of drip irrigation emitters to clogging caused by suspended sand particles. The results can accurately evaluate the combination of size and concentration of particles that caused clogging in each drip irrigation emitter, revealing trends in the sensitivity of emitters to clogging. Some scholars combined numerical simulations and PIV technology to analyze the influence of motion trajectory, velocity, particle size and content distribution of sand particles in the emitter on anti-plugging performance [15,16]. Lv et al. [17] conducted drip irrigation experiments under different combinations of sand concentration and operation time. The anti-clogging performance of drip irrigation emitters gradually decreased with the increase of channel length. Zhang et al. [18] altered the interdental structure and vortex zone within and increased the ability of water flow to carry sediment. Other researchers have suggested eliminating the low-speed and vortex regions. The stagnant flow zone was the direct cause of sediment deposition [19,20]. A small portion of research has found that the vortex area in drip irrigation emitters could increase energy dissipation and promote sand flow [21,22]. The key to solving blockage problems was optimizing the channel structure and revealing the motion characteristics of sand particles [23]. Orhan et al. [24] used CFD-DEM to simulate the three-dimensional fluid particle interaction process. This method provides a more intuitive representation of the trajectory and output rate of sand particles compared to the analysis of the flow field. The efficiency and accuracy of simulation were effectively improved.

The torus-margo structure in plant xylem achieves low energy consumption and pressure balance by dynamically adjusting the position of the pore membrane [25]. The torus-margo bordered pit structure is applied to the design of the flow channel structure of drip irrigation emitters. Xu et al. [26] designed a segmented flow channel leaf vein drip irrigation device based on the leaf vein structure of camphor trees. The actual test results showed that it has well hydraulic performance. Li et al. [27] optimized the flow channel structure of leaf vein drip irrigation emitters using genetic algorithms, further improving their anti-clogging performance. The pit bionic drip irrigation device in this study further combines the pressure adaptive characteristics and energy dissipation mechanism of the grooved structure. Liu et al. [28] developed the drip irrigation emitter that combines the reverse curvature of fig leaves with scaling laws for drip volume, which ensuring water-saving benefits and increasing crop yields. According to Li et al. [29] optimization design of the flow velocity in the mainstream area. The baffle structure was designed. The boundary structure and corners of the channel were changed to approximate a trapezoidal shape in order to improve its ability to use water flow to clean sand particles at corners. The channel width, depth and other dimensions were designed based on the proportional relationship of plant pore structure.

The current studies rarely explore the interplay between emitter structure optimization, internal energy dynamics and sand particle movement. In biomimetic design, only structural parameter design and water flow characteristics are considered. There is a lack of description of sand particle motion characteristics and actual anti-clogging experimental verification in the research process. The result cannot determine whether it can be used for actual irrigation. This study aims to: (1) The sand particle trajectories with velocity distributions was correlated; (2) The turbulence kinetic energy (TKE) in four bionic pit emitter designs were analyzed; (3) The optimal structure for hydraulic and anti-clogging performance was identified, then refined it by minimizing low-velocity zones and vortices; (4) The model was improved through numerical simulation and blockage testing. The findings offer theoretical guidance for developing high-efficiency, anti-clogging emitters.

## Materials and methods

### Physical model

**Fig 1:**
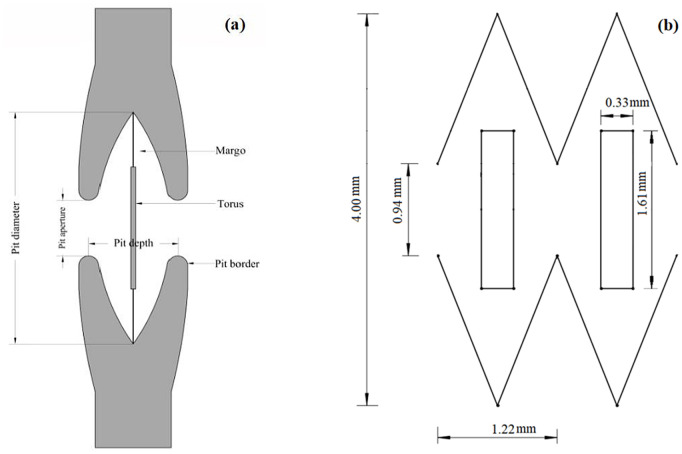
Bionic design of pit flow channel structure (a) Schematic of torus–margo bordered pit (b) Schematic of flow channel unit.

**Fig 2:**
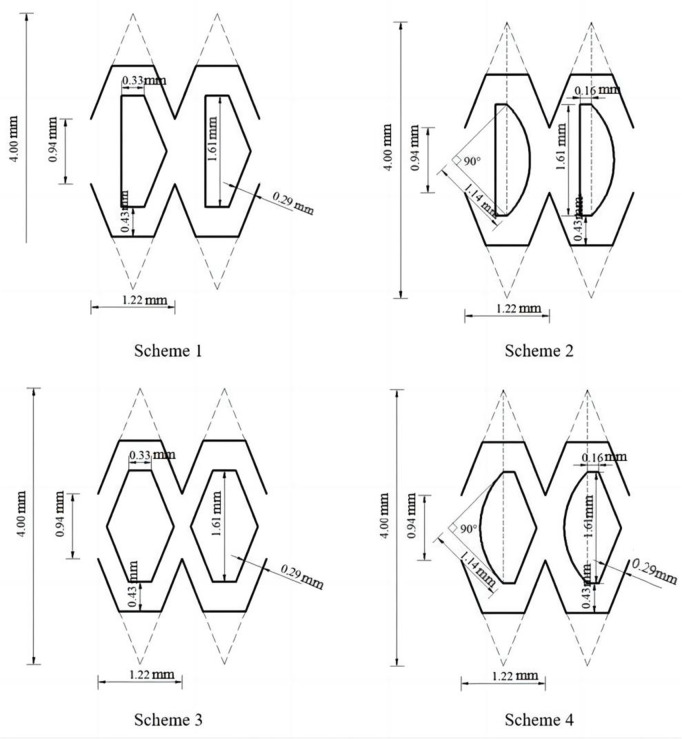
Schematic diagram of structural parameters of four different flow channels.

Tracheids are one of the main water transport methods in plant xylem. Water transport mainly depends on the pit structure between adjacent tracheids, which is composed of tens to hundreds of pit structures. Therefore, the torus–margo bordered pit structure in plant xylem tracheids has good pressure drop stability (Fig 1a). The pit bionic drip irrigation emitter is a new type of drip irrigation emitter constructed with the torus–margo bordered pit structure as the biomimetic structure (Fig 1b) [30]. According to the structural characteristics of drip irrigation emitters, four kinds of pit bionic drip irrigation emitters with different structures were designed [31]. Factors such as the cross-sectional area and length of the flow channel affected the hydraulic performance and anti-clogging ability of the drip irrigation emitter. The control variables were adopted to reduce the experimental errors. The other structures remain the same, changing the internal structure of the flow channel. The number of flow channel units was fixed at 12. The depth of the flow channel was set to 0.6 mm. The distance between the intersection points of the flow channel outer boundary extension lines was fixed at 4 mm. The length of each flow channel unit was fixed at 1.22 mm. The inlet and outlet of the flow channel were both 0.94 mm [1]. The structural parameters of four flow channels are shown in Fig 2.

### Grid division and numerical simulation

This study was conducted in 3D and meshing and numerical simulations were carried out by using COMSOL software. The standard *k-ε* turbulence model was adopted in the fluid domain of the pit bionic drip irrigation emitter. The inlet pressure was set at 50 kPa and the outlet of the flow channel was set as the outflow boundary. The boundary condition of the particles at the outlet was set to “disappear” and the boundary condition of the side wall was set to “rebound”. The same number of particles are released at the entrance according to the time step and their release location is random. The total number of particles was about 10,000. The particle density was 2500 kg/m^3^ and the particle size was 0.1 mm. The Poisson’s ratio was 0.4. The Shear modulus was 7.143 × 10^6^ N/m^2^. The Young’s modulus was 2 × 10^7^ N/m^2^. The Recovery coefficient was 0.5. The Rolling friction coefficient is 0.3. The Sliding friction coefficient is 0.01 [32,33]. The effects of viscous drag and gravity were considered in the simulation analysis. The effects of pressure gradient forces, virtual mass forces. The Saffman forces were not taken into account (small order of magnitude [34]).

The condition of no slip was applicable to the wall of the pit flow channel. In addition, a stochastic particle trajectory model (Euler–Lagrange two-phase flow model) was applied in the simulation. The continuous randomized wandering model was used. The water in the flow channel was regarded as an incompressible continuous fluid and the sand particles as discrete phases. As particle diffusion was caused by the fluctuation of a turbulent fluid, the simple algorithm was used to solve it. The main control equations for numerical simulation of the fluid domain include:

Continuity equation:


$$\frac{\partial \rho }{\partial t}+\frac{\partial }{\partial z_{i}}(\rho u_{i})=0$$ (1)

Momentum equation:


$$\frac{\partial }{\partial t}(\rho u_{i})+\frac{\partial }{\partial x_{j}}(\rho u_{i}u_{j})=-\frac{\partial p}{\partial x_{i}}+\frac{\partial }{\partial x_{j}}(\mu \frac{\partial u_{i}}{z_{j}}-\rho \overset{―}{u_{i}^{\prime }u_{j}^{\prime }})$$ (2)

where ρ is the density of the liquid, kg/m^3^; ui, uj is the average flow rate of the liquid, m/s; *µ* is the dynamic viscosity of the liquid, Pa·s; p is the average pressure of the liquid; i, j take the values of the range of 1, 2, 3.

Phase continuity equation of sand particles:


$$\frac{\partial {\overset{―}{\alpha }}_{d}}{\partial t}+\frac{\partial }{\partial \chi_{j}}({\overset{―}{\alpha }}_{d}{\tilde{v}}_{di})=0$$ (3)

Where ${\overset{―}{\alpha }}_{d}$ is volume fraction of sand particle phase; ${\tilde{v}}_{di}$ is viscosity coefficient of sand particle phase.

Normal force be-tween sand particles:


$$F_{n}=\frac{\text{4}}{\text{3}}E^{*}{R^{*}}^{\frac{\text{1}}{\text{2}}}k^{\frac{\text{3}}{\text{2}}}$$ (4)

Where E^*^ is equivalent modulus of elasticity; R^*^ is equivalent gravel radius.

Normal nylon resistance between sand grains:


$$F_{n}^{d}=-2\sqrt{\frac{\text{5}}{\text{6}}}\beta \sqrt{S_{n}m^{*}v^{rel}}$$ (5a)

$$m^{*}=\frac{m_{1}m_{2}}{m_{1}+m_{2}}$$ (5b)

Where m^*^ is equivalent mass; *β* is drag coefficient; *S*_n_ is normal stiffness; *v*^rel^ relative velocity; m_1_ mass of sand grain 1; m_2_ mass of sand grain 2.

Tangential force between sand particles:


$$F_{t}=-S_{t}\delta$$ (6a)

$$S_{t}=8G^{*}\sqrt{R^{*}k}$$ (6b)

$$G^{*}=\frac{2-\nu_{1}^{2}}{G_{1}}+\frac{2-\nu_{2}^{2}}{G_{2}}$$ (6c)

Where δ is tangential overlap; S_t_ is tangential stiffness; G^*^ is Equivalent shear modulus; G_1_ is shear modulus of sand grain 1; G_2_ is shear modulus of sand grain 2.

Tangential nylon resistance between sand grains:


$$F_{t}^{d}=-2\sqrt{\frac{\text{5}}{\text{6}}}\beta \sqrt{S_{t}m^{*}\nu_{t}^{rel}}$$ (7)

Where *v*_*t*_^rel^ is tangential relative velocity.

Sliding friction:


$$T_{i}=-\mu_{r}F_{n}R_{i}\omega_{i}$$ (8)

Where *µ* is Roling firiction factor; R_i_ is distance from the center of mass to the point of contact; ω_i_ is unit angular velocity vector of the object at the contact point.

There were still some limitations in the parameter settings for fluids and sand particles. Newtonian fluids were suitable for simple solutions with low concentration and low viscosity. The high concentration solution exhibits shear thinning characteristics and leads to an increase in viscosity error. The assumption of single density would ignore the actual density distribution of sand particles. The suspension capacity of lightweight particles was underestimated, such as sand particles encapsulating organic matter. This would affect its sedimentation rate.

**Fig 3:**
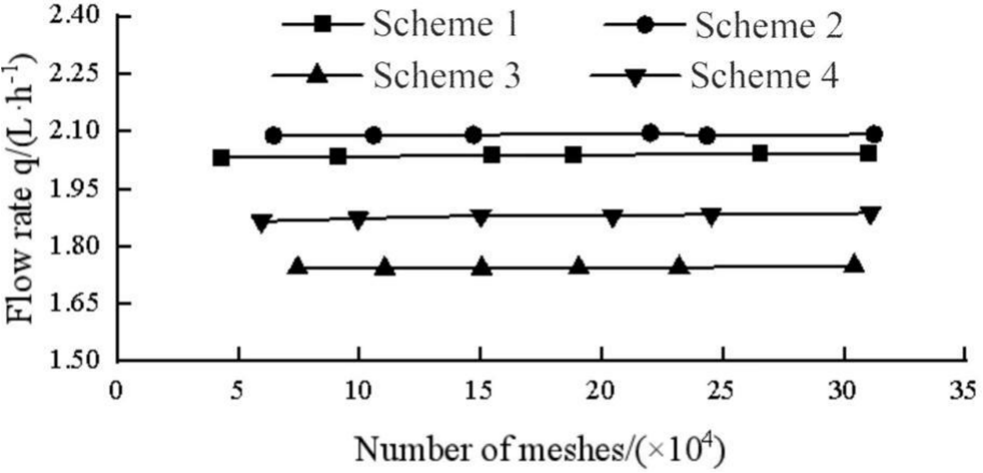
Grid independence test.

**Fig 4:**
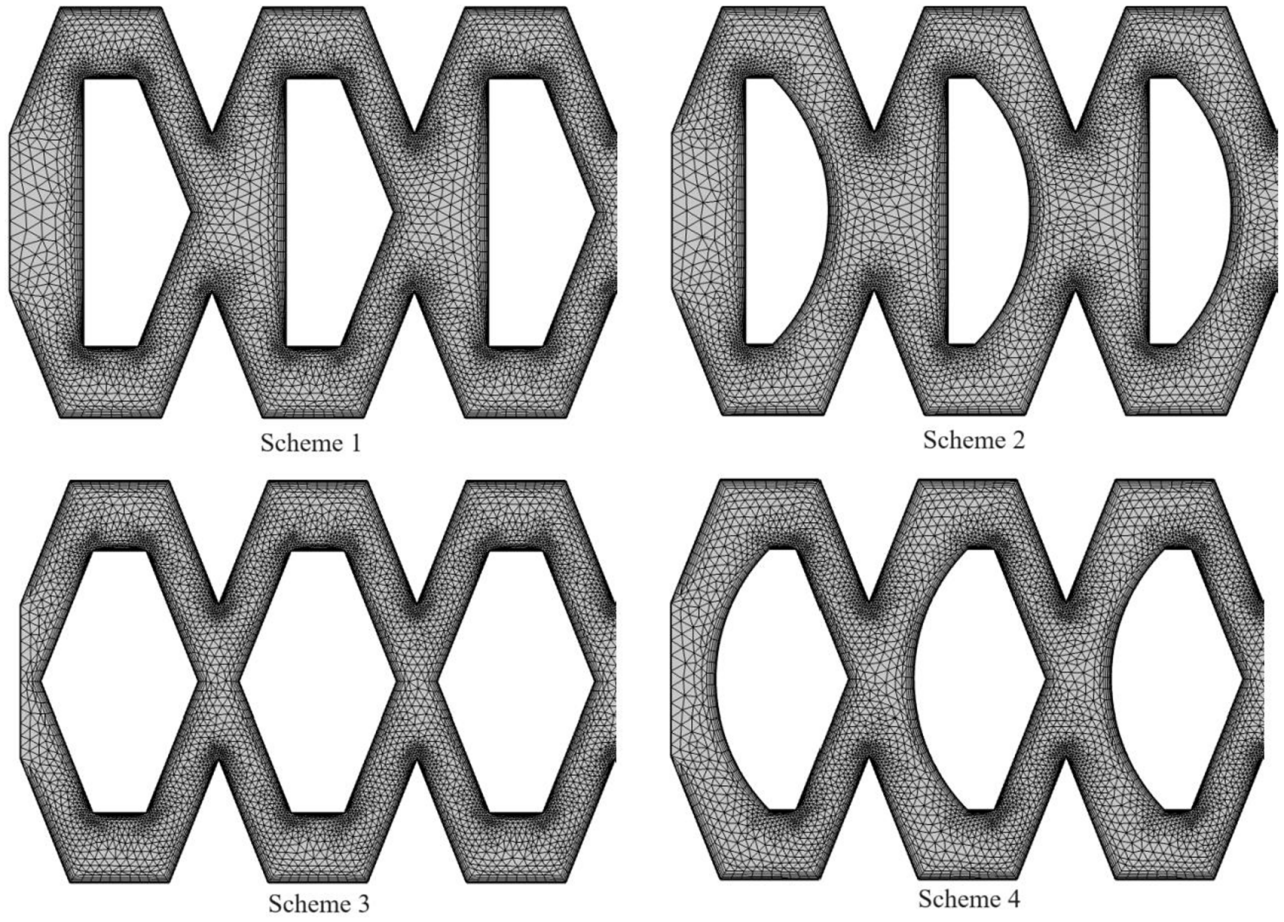
The grid division of emitter flow channel structures.

The fluid domain of the pit bionic drip irrigation emitter was divided by unstructured meshes of tetrahedrons and hexahedrons. The boundary layer mesh needed to be refined accordingly near the wall and a certain mesh refinement was performed at the corners of the flow channel structure. The total number of grids ranged from 200,000–500,000. The prediction accuracy of the inlet and outlet flow rate of the pit bionic drip irrigation emitter was tested by the grid independence (Fig 3). When the number of grids was greater than or equal to 200,000, the flow rate tended to stabilize. The total number of meshes would increase after encrypting the boundary layer mesh. An excessive number of grids could lead to a decrease in simulation experiment efficiency. This article selected a grid size of around 300,000 for relevant numerical simulation, the mean difference calculated at this point is less than 0.5% [35], in which the mesh size was 0.0576 mm at the maximum and 0.00864 mm at the minimum. The more detailed mesh division was carried out for the boundary layer. The mesh refinement near the walls mainly adopted the sweeping method. The hexahedron was mainly used in the mesh division, the tetrahedron was used as a supplement [36]. The minimum size of the boundary layer mesh was 0.00741 mm. The maximum surface size was 0.00821. The average orthogonal mass was 0.8158 and the minimum orthogonal mass was 0.91. The average aspect ratio was 2.4 and the maximum aspect ratio was 2.6. The maximum skewness was 0.28. The maximum value of y+ on the wall of the mesh was 49. According to the wall function method, the maximum value of y + was required to be between 30 and 300 [37]. The fluid domain grid division is shown in Fig 4.

### Test methods

**Fig 5:**
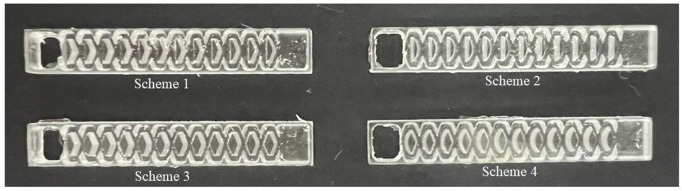
Physical picture of flow channel of emitter.

**Fig 6:**
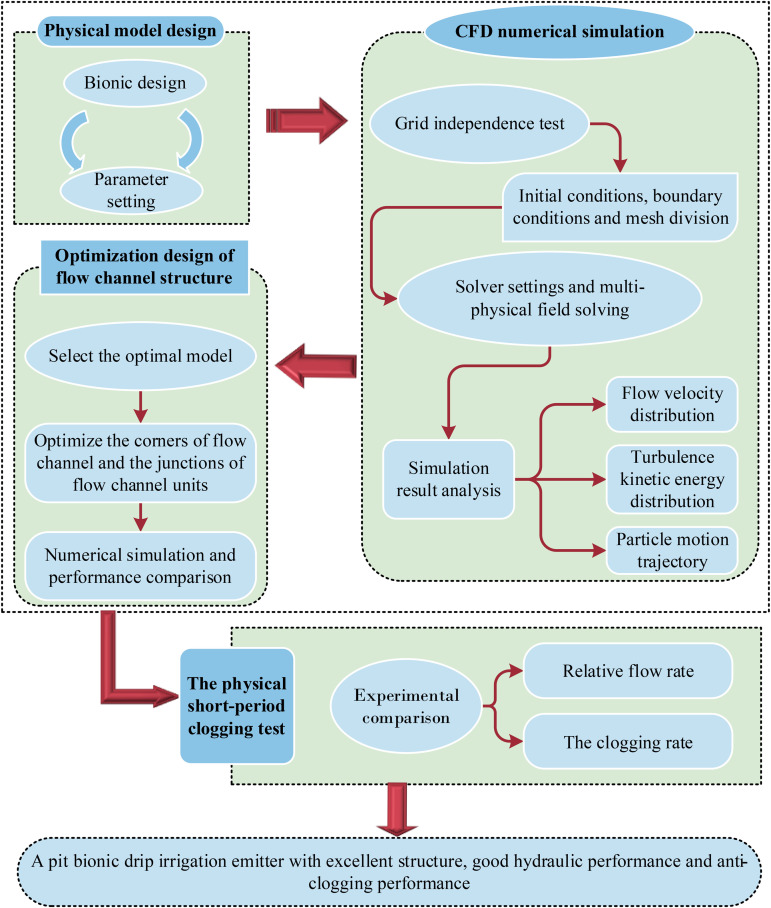
The details of flow chart.

**Fig 7:**
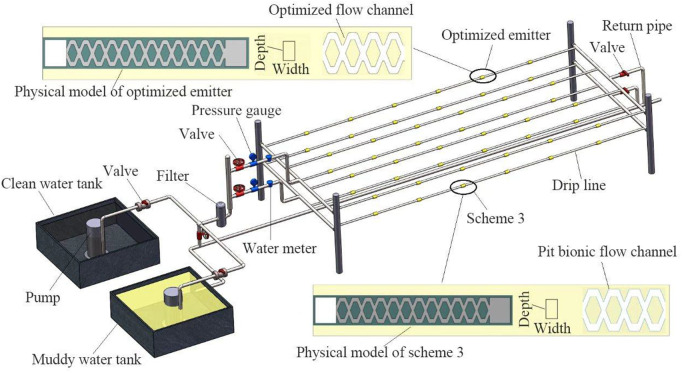
The physical short-period clogging test device.

In this study, SolidWorks software was used to design the test model. The EM-G32S-X32 high-precision engraving machine with a manufacturing accuracy of 0.01 mm and a repeatability accuracy of 0.005 mm was used to produce an equal-scale pit bionic drip irrigation emitter. The model was made of Plexiglas. Four kinds of emitter flow channels are shown in Fig 5. The detailed flow chart of this article is shown in Fig 6. The optimal performance scheme will be experimentally compared with the optimized emitter. The experimental device is shown in Fig 7. The experimental device was divided into two layers and each layer was set with four drip irrigation pipes. Each pipeline was equipped with six pit bionic drip irrigation emitters, totaling forty-eight pit bionic drip irrigation emitters. First, the flow rate of the drip irrigation emitter was tested with clean water under 50 kPa working pressure. Each test lasted for 5 minutes. The test was repeated 3 times. The emitter discharge of each test was recorded with a measuring cylinder, and the average value was taken as the rated flow rate. Referring to the standard of “Test Procedure for Short Period Clogging of Drip Irrigation Emitters” as explained in the literature [38], the test in this study was divided into eight stages. From the first stage to the eighth stage, different graded sand particles were gradually added to the water. The particle size gradation and concentration of sand particles are shown in Table 1. The sand particle size ranges from F60 to F220, which denotes screened sand particles from a 60 mesh to a 220 mesh. The clogging test was carried out on all the pit bionic drip irrigation emitters under 50 kPa working pressure. Different grades of sand particles were gradually added. The water tank was equipped with an automatic mixing device and a return pipe was installed at the end of the drip irrigation system to ensure uniform sand concentration. During the experiment, an electronic pressure gauge was used to continuously measure and adjust the pressure to ensure flow stability. The test was conducted every 24 hours in order to meet the actual working conditions of drip irrigation emitters. All tests were conducted using a graduated cylinder to measure the flow rate of drip irrigation emitters within 5 minutes. The average value was taken after three repeated measurements.

**Table 1:** Particle size gradations and concentrations of sand particles.

Stage	Particle size gradations/(mm)								Total concentration (mg/L)
	F220 ≤0.066	F180 0.083	F150 0.106	F120 0.120	F100 0.165	F80 0.187	F70 0.212	F60 0.245	
Stage 1	250	—	—	—	—	—	—	—	250
Stage 2	250	250	—	—	—	—	—	—	500
Stage 3	250	250	250	—	—	—	—	—	750
Stage 4	250	250	250	250	—	—	—	—	1000
Stage 5	250	250	250	250	250	—	—	—	1250
Stage 6	250	250	250	250	250	250	—	—	1500
Stage 7	250	250	250	250	250	250	250	—	1750
Stage 8	250	250	250	250	250	250	250	250	2000

The relative flow rate is the percentage of the average flow rate of all emitters to the rated flow rate, and the clogging rate is the percentage of the number of emitters with blockage to the total number of emitters. In this study, when the relative flow rate is less than 75% or the clogging rate is greater than 25%, it is considered that the pit bionic drip irrigation emitter is clogged; on the contrary, it is considered that the pit bionic drip irrigation emitter is normal. The relative flow rate and clogging rate can be expressed by equations (9) and (10).


$$K=\frac{{\bar{q}}_{i}}{Q_{0}}\times 100\%$$ (9)

$$A=\frac{\sum {\mathrm{\backslash nolimits}}_{j=1}^{M}I\left(q_{i,j}<0.75Q_{0,j}\right)}{M}\times 100\%$$ (10)

Where *K* was the relative flow rate; *q*_*i*_ was the average flow rate of all not blocked emitters in the i-th stage, L/h; *Q*_*0*_ was the rated flow rate of a single emitter in a clear water state, L/h; *A* was the clogging rate; *q*_*i,j*_ was the measured flow rate of the j-th drip irrigation emitter in the i-th stage; *Q*_*0,j*_ was the rated flow rate of the j-th sprinkler; *I* was the indicator function (taking 1 when conditions were met). *M* was the number of total pit bionic drip irrigation emitters.

## Results

### The velocity distribution inside drip irrigation emitter

**Fig 8:**
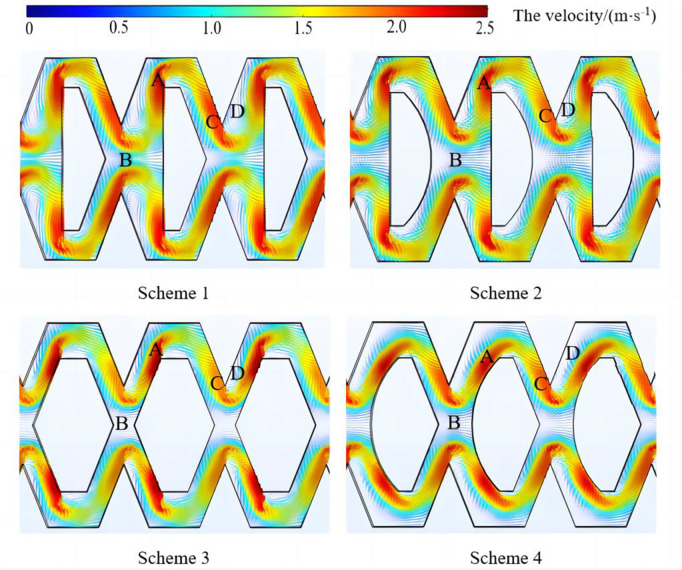
The velocity line contour distribution in the flow channels of the emitters.

The velocity line contour distribution at various positions inside the four types of pit flow channels under a working pressure of 50 kPa was shown in Fig 8. The fluid velocities at all points in the flow channel are not the absolute velocity of the pit drip irrigation emitter. However, the relative velocities at different locations in the flow channel are valid. The flow velocity in the flow channel and the area occupied by low flow velocity areas have a great influence on the anti-clogging performance of drip irrigation emitters.

As shown in Fig 8, according to the flow velocity distribution characteristics, the velocity line contour distribution inside each flow channel is divided into four regions: the main flow region (region A), the convection region (region B), the water-facing region (region C) and the backwater region (region D). Among them, both the main flow region and the water-facing region belong to the high-velocity region, and the velocity is generally higher than that of the convection region and the backwater region. This study focused on the flow velocity distribution in the low flow velocity region (convection region and backwater region). The velocity ranges of low-velocity regions inside the four flow channels are 0.0017 ~ 1.15m/s, 0.0051 ~ 1.02m/s, 0.0040 ~ 1.07m/s and 0.0043 ~ 0.98m/s. The maximum velocity of scheme 1 and scheme 3 is greater than that of scheme 2 and scheme 4, and the minimum velocity is smaller than that of scheme 2 and scheme 4. The area occupied by the low flow velocity zone in scheme 2 and scheme 4 is larger than that in scheme 1 and scheme 3, which increases the probability of sand particle retention. The low-speed areas would generate vortices and sand particles were prone to circulating within the vortices. The collision between sand particles and walls leads to a decrease in energy [39]. The centrifugal force of sand particles would decrease and it would be difficult for them to leave the vortex zone, which caused blockage of the pit bionic drip irrigation emitter. Scheme 1, scheme 2 and scheme 4 all have obvious low-velocity vortices in the backwater region (region D), while scheme 3 has a relatively light situation. The average velocity of Scheme 3 in the low-speed region (0.58m/s) was 81% higher than Scheme 1 (0.32m/s). The proportion of vortex area was 12% and the vortex was not very coherent, which promoted particle escape. The sand particles with low velocity are difficult to escape the eddy current and remain here, leading to further exacerbation of sand particle accumulation, which seriously affects the anti-clogging ability of the pit bionic drip irrigation emitter. Scheme 3 has a smaller proportion of low-speed vortex areas compared to common drip irrigation emitters on the market. Therefore, the scheme 3 design had certain theoretical advantages in improving the anti-clogging performance of the channel.

### Turbulence kinetic energy distribution in drip irrigation emitter

The change in total turbulence kinetic energy over time reflects the net budget of turbulence kinetic energy, which was an indicator to measure the development or decline of turbulence. The turbulence kinetic energy is directly proportional to the time-averaged flow velocity and turbulence intensity. There was a complex nonlinear relationship between the mean velocity and the turbulent kinetic energy; when the fluid velocity increases, the turbulent kinetic energy increases accordingly, and the interactions between the fluid particles become more violent, resulting in more turbulent motions. The ability of the fluid to displace sand particles increases. This reduces the probability of sand particles remaining in the flow channel and also means that the emitter has stronger anti-clogging ability.

**Fig 9:**
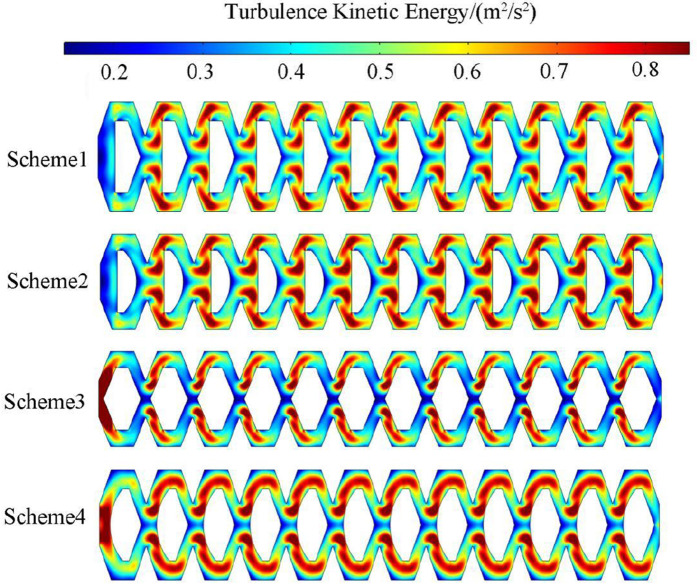
The turbulence kinetic energy distribution in the flow channel of emitter.

The turbulence kinetic energy distribution at various positions inside the four types of pit flow channels under a working pressure of 50 kPa is shown in Fig 9. In the same flow channel of the pit bionic drip irrigation emitter, the turbulence kinetic energy distribution characteristics of each flow channel unit after the first flow channel unit at the inlet are basically the same. The variation gradient of turbulence kinetic energy along the horizontal axis is small, but the variation gradient along the vertical axis is relatively large. The range of turbulence kinetic energy in four schemes is 0.046 ~ 0.471m^2^/s^2^, 0.045 ~ 0.561m^2^/s^2^, 0.117 ~ 0.940m^2^/s^2^ and 0.115 ~ 0.701m^2^/s^2^.

The minimum turbulence kinetic energy of scheme 3 and scheme 4 was relatively large. The scheme 3 and scheme 4 had greater energy to carry the migration of sand particles, which means that the probability of sand particle retention is lower. Scheme 1, scheme 2 and scheme 4 have lower turbulence kinetic energy at the inlet of the flow channel, which is easy to make a large number of sand particles to accumulate and cause serious blockage, leading to the decline of the anti-clogging ability of the pit bionic drip irrigation emitter.

When the fluid passed through the inclined baffle, the strong gradient was formed between the high-speed and low-speed zones and the shear layer became unstable. The instability of the shear layer led to the periodic generation and shedding of vortices and enhanced turbulence kinetic energy. The inlet cross-sectional area ratio of scheme 3 was reduced, which resulted in an increase in Reynolds number. The transition of water flow to turbulent state was promoted. Scheme 3 had a large turbulence kinetic energy at the inlet and the minimum turbulence kinetic energy was the largest. The probability of blockage in the flow channel at the inlet was reduced. The scheme 3 had better anti-clogging ability.

### The analysis of motion characteristics of sand particles

**Fig 10:**
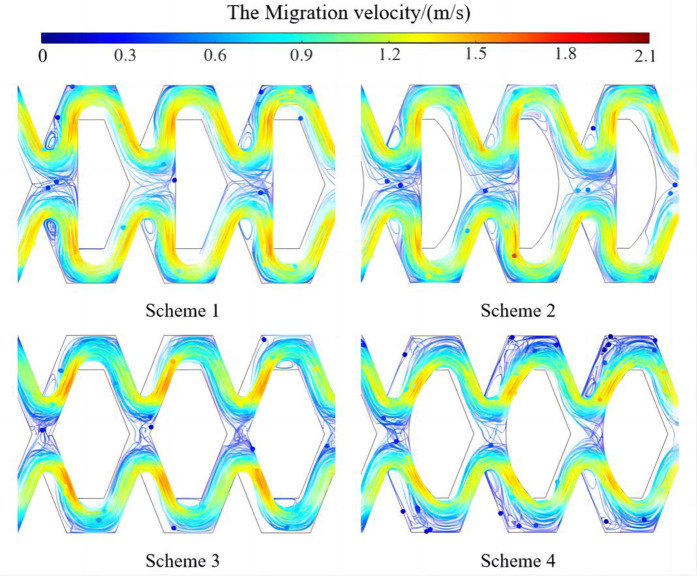
The Particle motion trajectory in the flow channel of emitter.

A certain amount of the same sand particles was released at the inlet of four different flow channels. The motion trajectories of sand particles inside the flow channels are shown in Fig 10. The motion trajectories of sand particles in four different flow channels were relatively consistent, and there was a clear relationship between them and the distribution of the water flow velocity field. Most of the sand particles followed the water flow of the high velocity region, and a small part of the sand particles moved from the high velocity region to the low velocity region. The sand particles tended to have irregular vortex motion in the backwater region (low-velocity region) and returned to the main flow region under the action of centrifugal force at the boundary. After sand particles entered the backwater region, the more turnover times they had, the longer they stayed here, and the more likely they were to accumulate, increasing the probability of blockage. The movement direction of a small number of sand particles in the convection region (low-velocity region) had a large deviation, and the sand particles had staggered movement in the upper and lower flow channels. Sand particles migrated irregularly at a low velocity in the convection region and collided with the internal baffles, which also led to the increase of particle retention time. With the continuous entry of subsequent particles, the accumulation situation would be aggravated, which led to the clogging of the emitter. This phenomenon was more obvious in scheme 1 and scheme 2, while scheme 3 and scheme 4 were relatively better.

### The optimization of flow channel structure

The structural parameters of the flow channel are the key factors to determine the hydraulic performance of the pit bionic drip irrigation emitter, and they have a great influence on the velocity, pressure, distribution of sand particles and the magnitude of turbulence kinetic energy inside the flow channel. Adjusting the structural parameters of the flow channel can increase the turbulence kinetic energy and improve the distribution of sand particles in the flow channel, which helps to further improve the anti-clogging performance of the pit bionic drip irrigation emitter. In this study, scheme 3 has better hydraulic performance and anti-clogging ability, so the further optimization design of the flow channel structure is carried out on the basis of scheme 3. The right angle has been changed to a rounded corner with a radius of 0.2 mm and the tooth tip structure has been removed on the basis of the original channel structure.

**Fig 11:**
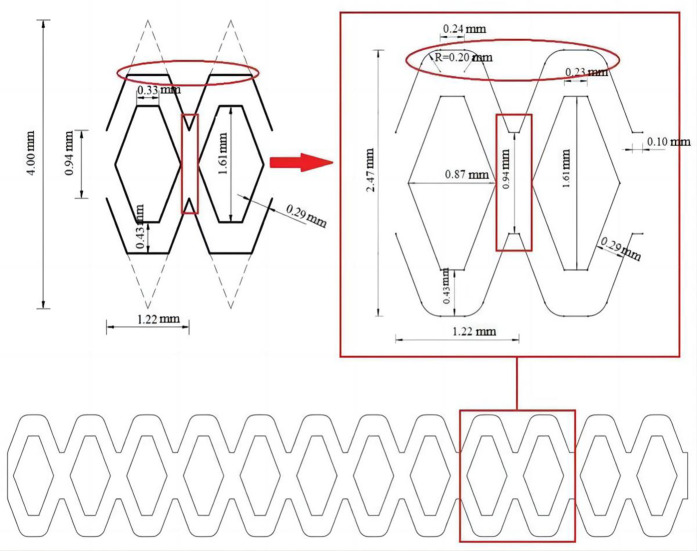
Schematic diagram of optimized channel structure and related parameters.

The optimized flow channel structure and related parameters are shown in Fig 11. The flow channel was only optimized at the corners of the flow channel and the junction of flow channel units. By changing the corners of the flow channel in scheme 3 from a right angle to a rounded angle, the area of the velocity stagnation region at the corner was reduced, which reduced the probability of accumulation at the corner and causing blockage. The tooth tip at the junction of the flow channel unit in scheme 3 was removed to increase the passage amount of time-uniform flow, which was beneficial for improving the minimum turbulence kinetic energy value in the low flow velocity region and improving the ability of sand particles to pass through the low flow velocity region. In this paper, relevant numerical simulation was carried out to analyze the turbulence kinetic energy distribution and the motion trajectory of sand particles in the optimized flow channel, and a comparative analysis was made with the above four kinds of flow channel.

**Fig 12:**
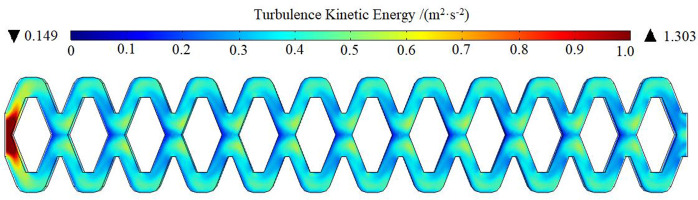
The turbulence kinetic energy distribution of optimized flow channel.

The turbulence kinetic energy distribution of the optimized flow channel is shown in Fig 12. The turbulence kinetic energy was obviously improved. The turbulence kinetic energy range of the optimized flow channel was 0.149 ~ 1.303m^2^/s^2^. The optimized scenarios show increases of 177% ~ 224%, 132% ~ 231%, 27% ~ 39%, and 30% ~ 86% compared to Scenarios 1, 2, 3, and 4. The improvement of turbulence kinetic energy was conducive to enhancing the stability of turbulence development, which facilitated the water flow to carry sand particles out better and reduced the probability of sand particles staying in the flow channel. Meanwhile, it also meant that the emitter had a stronger anti-clogging ability.

**Fig 13:**
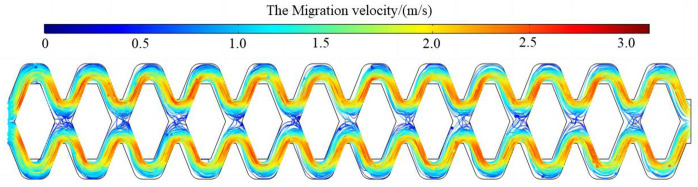
The Particle motion trajectory of optimized flow channel.

The motion trajectory of sand particles in the optimized flow channel is shown in Fig 13. The overall transport of sand particles was very smooth. No relatively complete low-velocity vortices were found in the low-velocity areas such as the backwater region and the corner of the flow channel, indicating a decrease in the binding force of the low-velocity area on sand particles, which was conducive to the movement of sand particles. Increasing the area of the low-velocity region at the junction of flow channel units (convection region) was conducive to the movement of sand particles. But there were still a few sand particles in random motion. The collision and sharp rotation of sand particles at the junction of flow channel units would cause a large amount of local water head loss, which improved the energy dissipation efficiency of the pit bionic drip irrigation emitter and was conducive to the stability of the outflow.

### The physical short-period clogging test

In order to verify whether the relevant simulation results are correct, short-period clogging experiments were carried out on scheme 3 and the optimized structure in this paper. Through observing the relative flow rate and clogging rate of the pit bionic drip irrigation emitter, the blockage was determined. The test results of relative flow rate are shown in Table 2. Stage 0 referred to the flow rate of two kinds of the pit bionic drip irrigation emitters in the clear water state. From the first stage to the eighth stage, sand particles of different sizes were gradually added into the water. The results showed that the relative flow rate of both flow channel structures decreased as the experimental stages progressed, indicating that the anti-clogging performance of the pit bionic drip irrigation emitter worsened with increasing sand particle concentration. The relative flow rate of scheme 3 was reduced by 2.34% ~ 23.71%, and the relative flow rate of the optimized emitter was reduced by 1.44% ~ 18.51%. Stage 4 has a higher relative flow than stage 3, and stage 7 has a higher relative flow than stage 6, possibly due to flushing action. The relative flow rate of two kinds of pit bionic drip irrigation emitters with different structures was greater than 75% at each stage, indicating that both of the two different emitters had not been blocked and had good anti-clogging ability. The relative flow rate of the optimized emitter was greater than that of scheme 3 at any stage. The relative flow rate increased 14.51% when the particle size was less than 0.12 mm.

**Table 2:** The test results of relative flow of emitters.

Type of emitter	Stage	Average flow/(L/h)	Relative flow/(%)	Type of emitter	Stage	Average flow/(L/h)	Relative flow/(%)
Scheme 3	0	1.775	/	Optimized emitter	0	1.945	/
	1	1.733	97.66		1	1.917	98.56
	2	1.636	92.16		2	1.904	97.91
	3	1.487	83.78		3	1.889	97.12
	4	1.475	83.12		4	1.899	97.63
	5	1.469	82.74		5	1.742	89.57
	6	1.482	83.47		6	1.716	88.24
	7	1.399	78.83		7	1.728	88.86
	8	1.354	76.29		8	1.585	81.49

**Fig 14:**
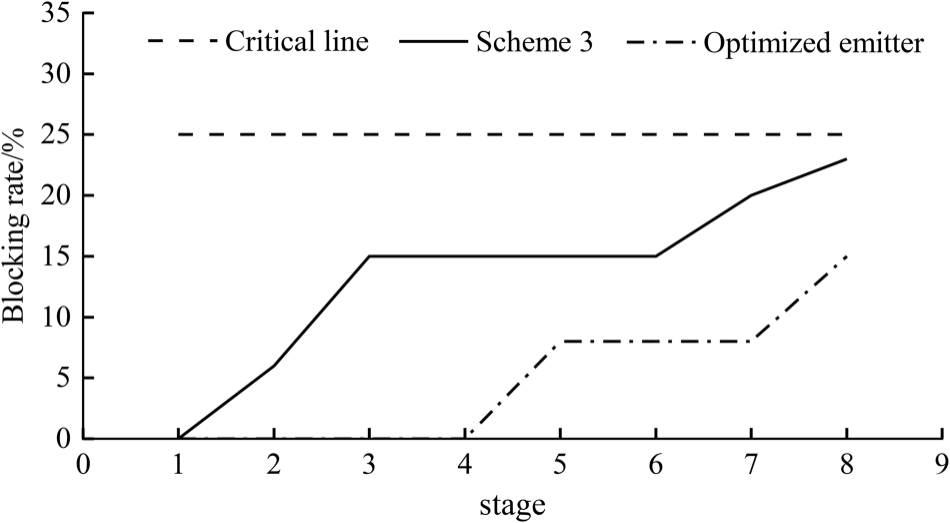
The short cycle blockage experimental test results.

The anti-clogging performance of two kinds of pit bionic drip irrigation emitters is shown in Fig 14. The blockage rates of Scheme 3 and optimized flow channel under eight different particle sizes of drip irrigation were all below the critical threshold. This indicated that if all eight types of sand particles were mixed, most of the sand particles will also pass through the entire channel.The particle size less than 0.083 mm will not cause blockage for scheme 3. The blockage state does not change when the particle size is between 0.106 ~ 0.187 mm. For the optimization plan, The particle size of 0.12 mm is required to cause blockage for the optimization scheme. When the particle size is 0.165 ~ 0.212 mm, the blockage state does not change and the blockage rate is lower than scheme 3.

There were multiple inflection points in the clogging rate of two drip irrigation emitters. The clogging rate of scheme 3 increased from the first stage to 15%, remained constant from the third to sixth stages, and finally climbed to 23% in the eighth stage. The optimized emitter did not experience blockage in the first to fourth stages, and the clogging rate increased to 8% in the fifth stage. The clogging rate remained constant from the fifth to seventh stages and finally reached 15% in the eighth stage. Among them, the clogging rate of two kinds of emitters had the biggest difference in the third and fourth stages, and the gap between the two reached 15%. By comparing the relative flow rate of two types of drip irrigation emitters in the third and fourth stages (as shown in Table 2). The relative flow rate of the optimized pit bionic drip irrigation emitter was 13.34% and 14.51% higher than that of scheme 3, when the particle size was less than 0.106 mm and 0.120 mm. The results showed that the anti-clogging performance of the optimized pit bionic drip irrigation emitter was greatly improved.

## Discussion

This article designed four types of pit bionic drip irrigation emitter structures. The flow velocity, turbulent energy and motion trajectory of sand particles in the flow channel were analyzed. The intrinsic relationship between the structure of pit bionic drip irrigation emitters and sand particle motion was revealed. It can be found that there was an obvious relationship between the motion trajectory of sand particles and the distribution characteristics of the water flow velocity field, which was basically similar to the research results of some scholars [40,41]. Scheme 1, Scheme 2, and Scheme 4 all had obvious low-velocity vortices in the backwater region. The total area of the low-velocity vortices was larger, which made it easier for sand particles to enter the low-speed zone. The collision resulted in energy loss and the centrifugal force of sand particles was relatively reduced between sand particles and walls. It was difficult for sand particles to leave the vortex zone. There were more sand particles gathering and settling in the vortex zone. The sand particles in Scheme 3 were more likely to pass through the entire flow channel. This was because the internal baffles of scheme 1 and scheme 2 were perpendicular to the direction of water flow, which had a certain blocking effect on the sand carried out by water flow and was not conducive to the movement of sand particles [42,43]. The internal baffle of Scheme 3 forms a certain angle with the direction of water flow, which reduced the energy consumption of water flow and promoted sediment transport. The sand particles collide and rapidly turn under the action of water flow and re-enter the main flow area along the direction of the baffle after irregular movement. The risk of sand sedimentation was reduced.

From the perspective of turbulence kinetic energy, the greater the turbulence kinetic energy, the more favorable the water flow carrying sand out. Water flow in the flow channel would cause a large amount of energy loss, mainly due to local head loss caused by sharp turns and collisions of water flow and sand particles in the process of movement, which was the same as the conclusion of Guo et al. [44] and Aswini et al. [45]. The maximum turbulence kinetic energy of schemes 1, 2 and 4 mainly appeared in the main flow region, near the corner of the flow channel, while the minimum turbulence kinetic energy occurred at the inlet of the flow channel, which was basically consistent with the conclusion drawn by Yang et al. [46]. On the contrary, scheme 3 showed that its maximum turbulence kinetic energy appeared at the inlet of the flow channel, while the minimum turbulence kinetic energy occurred at the front end of the convection region and the water-facing surface of the tooth tip. The reason was due to the different flow channel structure and related parameters. At the same time, the distance between the inlet of the flow channel and the internal baffle of scheme 1, scheme 2 and scheme 4 was relatively far, and the inlet of the flow channel showed a sudden expansion state, which led to the decrease of inlet velocity and turbulence kinetic energy. The distance between the inlet of the flow channel and the internal baffle in scheme 3 was relatively close, and the cross-sectional area of the flow channel was also smaller. According to the continuity equation, its velocity was higher than the other three schemes. The water flow would turn sharply and collide under the action of the internal baffle, which made the water flow more chaotic and the turbulence intensity greater. It indicated that it was easier to maintain or develop into a turbulent flow state, and that its turbulence kinetic energy was larger. The main reason for the lower turbulence kinetic energy of the front end of the convection region and the water-facing surface of the tooth tip was the lower water flow velocity or turbulence intensity.

The optimization method in this article combined some methods proposed by scholars [47,48]. The area of the velocity stagnation zone was reduced. The possibility of sand accumulation and blockage was reduced. The minimum turbulence energy in the low-speed zone was increased and ensured uniform energy distribution of the water flow in the channel. The relative flow rate of the optimized pit bionic drip irrigation emitter was greater than that of scheme 3 at any stage. The first blockage of scheme 3 occurred in the second stage (F180), where the highest sand particle size was 0.083 mm. The first blockage of the optimized flow channel occurred in the fifth stage (F100), where the highest sand particle size was 0.165 mm. In terms of the sensitive particle size causing blockage, the sedimentation rate was reduced by an average of 58.02%.

The actual drip irrigation effect in farmland may be affected by the following factors. The optimized flow channel improved the anti-clogging performance of these eight types of sand particles, while the anti-clogging performance of ultrafine particles (<0.05 mm) or coarse particles (>0.3 mm) may be weakened. Yao et al. [49] indicated that was related to the capture mechanism in the medium particle size vortex region. If high concentration fertilizer water is used, the dynamic viscosity in the water quality increases. The movement characteristics and TKE of sand particles were affected, which led to an increase in the sedimentation rate of sand particles. The water temperature below 15°C will also have the same effect. Large scale farmland drip irrigation used low-quality water. Ions in water undergo chemical reactions inside the flow channel, forming physical chemical blockages. Algae and microorganisms secrete viscous extracellular polymers to form biofilms and exacerbate complex blockages. Despite the excellent performance of the optimized flow channel under laboratory conditions, the following issues still need to be addressed for its industrialization. The rounded corners of the flow channel imposed high limitations on the manufacturing process. The use of high-precision instruments was required and increased manufacturing costs. When the sediment concentration in irrigation water was high, different purpose filters needed to be equipped. Long term operation required monitoring of flow fluctuations and timely cleaning work. The optimization conclusion of this study was based on short cycle anti-clogging experiments, without considering the corrosion effect of material aging and extreme water quality on the surface of the flow channel. These factors will be further analyzed in subsequent work through CFD-DEM coupled simulation and accelerated aging experiments.

## Conclusion

1) There was a close relationship between the motion trajectory of sand particles and the distribution of the water flow velocity field. When two streams of liquid with different speeds and directions collided with each other, the flow direction of the low-speed liquid changed, leading to the formation of vortex regions. Scheme 1, scheme 2 and scheme 4 all have obvious low-velocity vortices in the backwater region (region D), while scheme 3 has a relatively light situation. In the vortex zone with low velocity, where sand particles would undergo irregular vortex motion, increasing the probability of sand particle accumulation.
2) In unstable flows, the velocity field was subject to violent shifts and vortex structures, leading to irregular velocity variations and pulsation enhancement and causing an increase in turbulent kinetic energy. This increased the ability to transport gravel with liquids. There was a positive correlation between the anti-clogging performance of the pit bionic drip irrigation emitter and the magnitude of turbulence kinetic energy in a certain range.
3) The optimized pit bionic drip irrigation emitter reduced the area of velocity stagnation region at the corner. Compared with the four schemes before optimization, the turbulence kinetic energy has been greatly improved. In the eight stages of the short-period clogging test, the relative flow rate of the optimized emitter was greater than that of scheme 3 at any stage. The relative flow rate increased 14.51% when the particle size was less than 0.12 mm. When the particle size was 0.120, 0.165, 0.187, 0.212, and 0.245 mm, the sedimentation rate was reduced by an average of 58.02%.
4) The low flow velocity region is the main cause of clogging in drip irrigation emitters, and the low flow velocity vortex greatly increases the probability of solid particle accumulation. Therefore, in the subsequent research on physical clogging of drip irrigation emitters, we can focus on reducing the area of low flow velocity and increasing the area of the main stream, so as to improve the anti-clogging ability of drip irrigation emitters.
